# Editorial: Molecular modulators of GPCRs signal transduction

**DOI:** 10.3389/fendo.2023.1252734

**Published:** 2023-07-18

**Authors:** Milka Vrecl, Marialessandra Contino, Marcello Leopoldo

**Affiliations:** ^1^ Institute of Preclinical Sciences, Veterinary Faculty, University of Ljubljana, Ljubljana, Slovenia; ^2^ Dipartimento di Farmacia – Scienze del Farmaco, Università degli Studi di Bari Aldo Moro, Bari, Italy

**Keywords:** GPCRs, signal transduction, molecular modulators, probe design, oligomerization

G protein-coupled receptors (GPCRs) are membrane proteins that cells use to convert many extracellular signals (hormones, neurotransmitters, and light, to mention a few) into intracellular responses. The human genome encodes 850 GPCRs, half considered potential drug targets. Consequently, GPCRs are still considered an attractive drug target class, so GPCRs are the focus of constant research. In fact, over the last ten years, approximately one thousand papers per year have been published on the PubMed database dealing with GPCR research.

This Research Topic was dedicated to original research and review articles dealing with a broad range of relevance to the design, development, and biological evaluation of molecular modulators of GPCRs-mediated signaling and it was compiled in cooperation with the European Research Network on Signal Transduction (ERNEST COST Action). The papers tackled various aspects of GPCR research.

The study by Pawnikar and Miao focused on the search for peptides capable of activating CXCR4 chemokine receptor by developing a method that used a novel Peptide Gaussian accelerated molecular dynamics (Pep-GaMD) approach to explore representative binding conformations of each peptide and identify critical low-energy states of CXCR4 activated by the super versus partial peptide agonists.

The study by Saecker et al. dealt with a different relevant aspect of GPCR research, namely the recruitment of arrestins following the interaction of a GPCR with an agonist. Specifically, the authors developed a live cell assay for adenosine A1 receptor (A1AR)-mediated β-arrestin 2 recruitment based on NanoBit^®^ technology. The assay characterized a set of partial and full A1AR agonists with highly reproducible results and an excellent signal-to-noise ratio.

The study by Ma et al. pursued the development of a fluorescent probe for GPR120, a potential target for many physiological diseases, including type 2 diabetes mellitus. The authors identified compound D5 as a potent GPR120 agonist with high activity and selectivity *in vitro* and a significant glucose-lowering effect *in vivo*.

Finally, the review article by Farooq et al. provided a detailed overview of strategies and techniques that can be employed to target GPCR oligomerization. In fact, GPCRs do not only exist and function in their monomeric form but can form higher-order oligomers or dimers with other GPCRs or even other classes of receptors. GPCR oligomers can modulate the pharmacological responses of the receptors and, therefore, could have important functional roles in an array of diseases, including cancer and several neurological and neuropsychiatric disorders.

## Author contributions

ML wrote the draft of the editorial. All authors contributed to the manuscript revision, read, and approved the submitted version.

